# Role of Lipopolysaccharide in Protecting OmpT from Autoproteolysis during In Vitro Refolding

**DOI:** 10.3390/biom10060922

**Published:** 2020-06-18

**Authors:** Gaurav Sinsinbar, Sushanth Gudlur, Kevin J Metcalf, Milan Mrksich, Madhavan Nallani, Bo Liedberg

**Affiliations:** 1 Center for Biomimetic Sensor Science, School of Materials Science and Engineering, Nanyang Technological University, Singapore 637553, Singapore; gaurav011@e.ntu.edu.sg (G.S.); sgudlur@ntu.edu.sg (S.G.); MNallani@ntu.edu.sg (M.N.); 2 Departments of Chemistry and Biomedical Engineering, Northwestern University, Evanston, IL 60208, USA; kevin.metcalf@northwestern.edu (K.J.M.); milan.mrksich@northwestern.edu (M.M.)

**Keywords:** LPS, autoproteolysis, OmpT, heat modifiable protein, omptin family, outer membrane protease, *E. coli*

## Abstract

Outer membrane protease (OmpT) is a 33.5 kDa aspartyl protease that cleaves at dibasic sites and is thought to function as a defense mechanism for *E. coli* against cationic antimicrobial peptides secreted by the host immune system. Despite carrying three dibasic sites in its own sequence, there is no report of OmpT autoproteolysis in vivo. However, recombinant OmpT expressed in vitro as inclusion bodies has been reported to undergo autoproteolysis during the refolding step, thus resulting in an inactive protease. In this study, we monitor and compare levels of in vitro autoproteolysis of folded and unfolded OmpT and examine the role of lipopolysaccharide (LPS) in autoproteolysis. SDS-PAGE data indicate that it is only the unfolded OmpT that undergoes autoproteolysis while the folded OmpT remains protected and resistant to autoproteolysis. This selective susceptibility to autoproteolysis is intriguing. Previous studies suggest that LPS, a co-factor necessary for OmpT activity, may play a protective role in preventing autoproteolysis. However, data presented here confirm that LPS plays no such protective role in the case of unfolded OmpT. Furthermore, OmpT mutants designed to prevent LPS from binding to its putative LPS-binding motif still exhibited excellent protease activity, suggesting that the putative LPS-binding motif is of less importance for OmpT’s activity than previously proposed.

## 1. Introduction

Gram-negative bacteria have evolved several different strategies to evade or counter antimicrobial peptides (AMPs) secreted by the host immune system. One such strategy, adopted by the *Enterobacteriacea* family, involves recruiting omptin proteases to cleave AMPs and making them ineffective. Omptins are a family of outer membrane proteases that share 40–80% amino acid sequence similarity and a highly conserved active site [1]. Examples include OmpT (*Escherichia coli*) Pla (*Yersinia pestis*), PgtE (*Salmonella enterica*), IcsP (*Shigella flexneri)*, and CroP (*Citrobacter rodentium*) [1,2]. *E. coli* OmpT is the most notable omptin protease and is thought to play an essential role in the bacteria’s defense system as well as in their virulence [3,4,5]. It is overexpressed in uropathogenic *E. coli* (UPEC) associated with the majority of community-acquired urinary tract infections [6,7], making UPECs more resistant to AMPs than commensal *E. coli* strains [6]. Besides UPEC, OmpT expression is also upregulated in certain *E. coli* strains involved in foodborne infections [8,9], indicating an active role in the pathogenicity of *E. coli* strains. Among the two genetically related gastrointestinal pathogens—enterohemorrhagic *E. coli* (EHEC) and enteropathogenic *E. coli* (EPEC)—that cause severe diarrheal diseases in humans [10], OmpT overexpression in EHEC allows the active degradation of AMPs to promote bacterial survival [5,11,12,13].

**Scheme 1:**
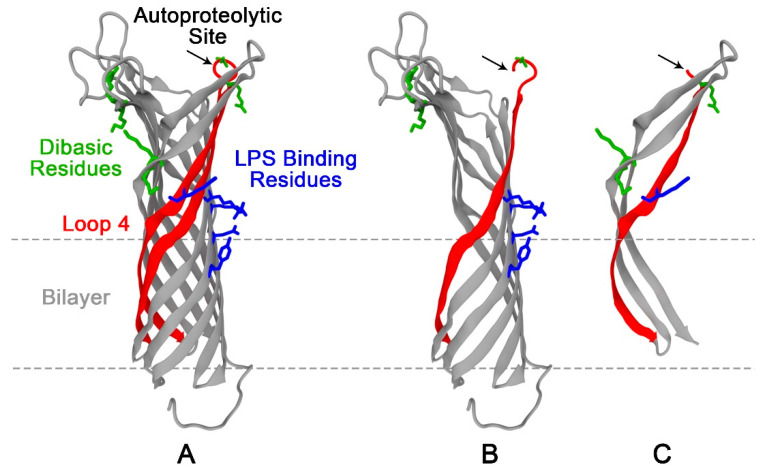
Structure of outer membrane protease (OmpT) and its autoproteolyzed fragments. (**A**) Ribbon representation of OmpT with the five predicted lipopolysaccharide (LPS) binding residues (blue) and the three dibasic sites represented in licorice (green). Black arrow points at the location of the dibasic site on loop 4 (red) that is prone to autoproteolysis yielding the two autoproteolytic fragments (**B**,**C**). Note: Crystal structure of wild type OmpT is not available. The Protein Data Bank (PDB) structure (1I78) of _mt_OmpT_DB_, an OmpT mutant (mt) that has one of its dibasic (DB) sites mutated (K217-R218 mutated to K216-G217-R218), is used here to illustrate the different key points, e.g., the locations of the autoproteolytic and predicted LPS binding residues.

OmpT is a 33.5 kDa membrane protein that adopts a β-barrel structure consisting of 10 antiparallel β-strands spanning the outer membrane with an active site facing the extracellular environment [1,3,14] (Scheme 1A). Previous work reports that OmpT requires lipopolysaccharide (LPS) as a co-factor for its proteolytic function [15]. However, the exact mechanism of how LPS activates OmpT is poorly understood. It is proposed that the binding of LPS to the putative binding motif in OmpT causes a slight conformational change in the protease’s active site which is associated with an active OmpT [15]. An active OmpT preferentially cleaves host-generated antimicrobial peptides at dibasic sites [15,16,17]. Despite carrying three dibasic sites in its own sequence, there is no report of OmpT autoproteolysis in vivo. The biogenesis of OmpT in vivo is a complex process starting with the cytosolic production of the protein that is translocated across the inner membrane through translocases in a partially folded state [18]. Upon entering the periplasmic space, the protein is fully folded and inserted into the outer membrane with the help of periplasmic chaperones and the β-barrel assembly machinery (BAM) complex [18]. Once in the outer membrane, OmpT becomes active, most likely when it binds to the LPS that is found only in the outer leaflet of Gram-negative bacteria. An inactive OmpT during its transport from the cytosol to the outer membrane possibly serves as a safety mechanism evolved to prevent any accidental proteolysis of cytosolic proteins.

A better understanding of OmpT autoproteolysis can help in designing antibiotics that induce and accelerate such detrimental events in vivo, thereby making the bacteria susceptible to host generated AMPs. Similarly, understanding LPS’s role in OmpT activity can help devise strategies to interfere or block LPS binding to OmpT, thus making the protease inactive and incapable of degrading host-generated AMPs that attack the bacteria. Most of what we know today about OmpT is based on in vitro studies of recombinant OmpT. In vitro expression of recombinant OmpT can be achieved either in the *E. coli* outer membrane or overexpressed as cytoplasmic protein aggregates called inclusion bodies (IBs) that are easily isolated and subsequently solubilized, refolded, and purified. The latter method is favored since it offers higher yields and ease of purification; however, OmpT undergoes autoproteolysis during in vitro refolding and purification resulting in an inactive protease [19] (Scheme 1B,C). Among its three dibasic sites (R_37_-K_38_, K_217_-R_218_, and K_259_-K_260_), only K_217_-R_218_ is prone to autoproteolysis [19]. Located on a flexible extracellular L4 loop on top of the beta-barrel structure of OmpT, this exposed site makes it more susceptible to autoproteolysis than the other two sites that are located on the body of the barrel (Scheme 1) [1]. Previously, autoproteolysis of recombinant OmpT during in vitro refolding was overcome via two different strategies. In the first strategy adopted by Kramer et al. (2000) [19], the protease sequence was mutated in the vicinity of the autoproteolysis site, K_217_-R_218_ was mutated to K_216_-G_217_-R_218_ [19]. However, this mutant was reported to have a lower activity (~30% lower) in comparison with the wild type protease [19]. In addition, we recently reported that this minor mutation in OmpT resulted in an altered substrate preference [20]. A second strategy, adopted by Wu et al. (2012) [21] and described in detail in Wu’s PhD thesis, involved the addition of LPS to refolded OmpT immediately after cation exchange purification to avoid autoproteolysis [21,22]. In this case, the author suspected a protective role of LPS against OmpT autoproteolysis, but did not characterize it any further.

In this study, we aim at gaining a deeper understanding of the in vitro conditions that promote autoproteolysis and the role of LPS in autoproteolysis. Using SDS-PAGE data, we demonstrate that a folded OmpT does not undergo autoproteolysis, but instead, it is the unfolded protein that undergoes proteolytic cleavage. Additionally, we show that LPS neither protects unfolded OmpT from autoproteolysis nor is its binding to the putative LPS-binding motif in OmpT essential for activity, as was previously thought, suggesting that there may be more than one allosteric LPS binding site on OmpT that is critical for its activity.

## 2. Materials and Methods

### 2.1. Materials

The plasmid ompT_pET28a was a gift from Prof. Neil Kelleher (Northwestern University, Evanston IL, USA) while plasmid mut_ompT_pET28a was synthesized and sequenced by Bio Basic Inc (Singapore). *E. coli* Rosetta™ (DE3) cells with T7 RNA polymerase under the *lac* promoter were used for expressing wild type and mutant OmpTs as IBs and were purchased from Merck Millipore (Singapore). LB Broth was purchased from BD Difco (Franklin Lakes, NJ, USA). Kanamycin was purchased from Gibco by Life technologies (Now, ThermoFisher Scientific, Waltham, MA, USA). Isopropyl thio-β-D-galactopyranoside (IPTG) was purchased from Gold Biotechnology (St. Louis, MO, USA). N-Dodecyl-N, N-dimethyl-3-ammonio-1-propanesulfonate (DodMe_2_NPrSO_3_) and lipopolysaccharides (LPS) from *E. coli* 0111:B4 purified by phenol extraction, Corning^®^ Black, flat bottom 384-well plates were purchased from Sigma Aldrich (Singapore). Urea was purchased from Affymetrix USB (Santa Clara, CA, USA). PD-10 Desalting Columns containing Sephadex G-25 resin were purchased from GE Healthcare Life Sciences (Singapore). HiPPR™ Detergent Removal Spin Column was purchased from ThermoFisher Scientific (Singapore). Synthetic peptide, Abz-ARRA-Tyr (NO_2_)-NH_2_, Ac-GARKVG-NH2, and Ac-GARIIG-NH2 were purchased as lyophilized powder and certified >95% purity from Biomatik (Cambridge, ON, Canada).

### 2.2. Recombinant OmpT Expression

The *ompT* gene corresponds to a polypeptide of 317 amino acids whose first 20 amino acids act as a signal peptide responsible for transferring OmpT into the periplasm [23]. The plasmid ompT_pET28a had a construct where the mature OmpT sequence corresponding to 297 amino acids in addition to methionine and alanine at the N-terminal was cloned without the signal peptide sequence between NcoI and EcoRI into the pET28a vector to produce ompT_pET28a. The N-terminal methionine, incorporated for initiation of the translation, is eventually excised upon expression [24]. The mutant *ompT* gene (G_216_K and K_217_G), which was synthesized and subcloned into the Xbal and Xhol site into the pET28a vector to produce mut_ompT_pET28a, was purchased from Bio Basic Inc. The wild type and mutant OmpTs (298 amino acids) were expressed as IBs in *E. coli* Rosetta™ (DE3) cells using the method previously described by Kramer, R.A. et al. (2000) [19]. Briefly, Rosetta™ (DE3) cells with ompT_pET28a and mut_ompT_pET28a plasmids were grown separately overnight in 5 mL LB Broth with 50 μg/mL kanamycin at 37 °C in a shaker set to 220 RPM. This primary culture was used to inoculate 200 mL LB Broth secondary cultures that were later induced with IPTG (1 mM final concentration) when the OD_600_ reached ~0.8–1. The cells were harvested after 4 h and washed with 50 mM Tris-HCl, pH 7.5 before suspending them in 5 mL lysis buffer (50 mM Tris-HCl, 40 mM EDTA, pH 8.0). These cells were kept on ice for 20 min before adding a further 5 mL of pre-chilled lysis buffer, followed by a second incubation on ice for 20 min. After treatment with the lysis buffer, the cells were sonicated for 7 min (2 s on, 4 s off, and 50% amplitude), upon which, the solution turned viscous. This viscous solution was centrifuged at 4500× *g* for 30 min to separate the IBs as pellets which were then washed twice with 30 mL wash buffer (10 mM Tris-HCl, 1 mM EDTA, pH 8.0). During the final wash, the solutions were aliquoted into separate tubes and centrifuged to obtain IBs as pellets.

### 2.3. Refolding of OmpT IBs

The IBs as pellets were solubilized in a solubilizing buffer (20 mM Tris-HCl, 8 M urea, 50 mM glycine, pH 8.3) and kept on ice for 1 h before the addition of pre-chilled refolding buffer (20 mM Tris-HCl, 31 mM DodMe_2_NPrSO_3_, pH 7.5) such that the final concentration of protein and urea was between 0.8 and 1 mg/mL and 0.8 M, respectively. The samples were kept on ice for 3–4 h. For samples requiring 4.5 mg/mL LPS during the refolding step, LPS was added to the refolding solution before mixing with the IBs in the solubilizing buffer. The refolded OmpT samples were centrifuged at 10,000× *g* for 10 min to pellet the protein aggregates. To remove the remaining urea from the samples, PD-10 columns were used. The columns were equilibrated with buffer (20 mM Tris, 10 mM DodMe_2_NPrSO_3_, pH 7.5). Refolded protein was loaded on the PD-10 column and fractions were collected. The fractions were checked for protein concentration and pooled together. Samples were aliquoted and stored at −20 °C for further use.

### 2.4. OmpT Protease Activity in the Presence of an Inhibitor

Based on a method previously described by Kramer et al. (2000) [19], OmpT IBs refolded in refolding buffer with 31 mM DodMe_2_NPrSO_3_ (RC-31), refolding buffer with 10 mM DodMe_2_NPrSO_3_ (RC-10), and refolding buffer with no DodMe_2_NPrSO_3_ (RC-0) were assayed for their protease activity in the presence of increasing concentrations of an inhibitor. All fluorescence assays were performed in 384-well plates at a final reaction volume of 100 μL. Synthetic peptide substrate, Abz-ARRA-Tyr (NO_2_)-NH_2_, is reported to be a good substrate for OmpT and has been used for determining OmpT activity [19]. The peptide substrate was prepared as stock solutions of 0.5 mM in Milli-Q water. For the assay, the peptide stock solution was diluted with a mixture of Milli-Q water and 10× assay buffer (200 mM Bis-tris, 20 mM DodMe_2_NPrSO_3_, 50 mM EDTA, pH 6.7) such that the final peptide concentration was 15 μM in 1× assay buffer (20 mM Bis-tris, 2 mM DodMe_2_NPrSO_3_, 5 mM EDTA, pH 6.7). The concentrations of all three OmpT samples were kept the same and confirmed using a NanoDrop^TM^ 2000c spectrophotometer (ThermoFischer Scientific, Singapore) and OmpT’s molar absorption coefficient of 74,960 M^−1^cm^−1^ [19]. A stock solution of 2 mg/mL of LPS from *E. coli* was prepared in water. All three samples of OmpT were diluted 10 times and incubated with 20 µg/mL LPS for ~15 min. An amount of 10 µL of OmpT-LPS mixture was mixed with 10 µL of ZnCl_2_ at different concentration (1000, 500, 250, 62.5, 15.6, 7.8, and 3.9 10 µM). An amount of 10 μL of this OmpT-LPS-ZnCl_2_ mixture was added to 90 μL of 15 μM peptide substrate in assay buffer. The enzymatic reaction was performed at 37 °C. The increase in fluorescence intensity upon OmpT addition was collected over time using a TECAN infinite^®^ M200 PRO (Männedorf, Switzerland) fluorescence spectrophotometer fitted with a plate reader. Fluorescence intensity signals were collected every 10 s for 60 min with the excitation wavelength set to 325 nm and emission at 420 nm, and the data plotted in OriginPro 9.1 (OriginLab Corporation, Northampton, MA, USA). The initial velocity of the enzymatic reaction (µMs^−1^) was calculated using the data points in the linear range of the kinetic data. The reaction velocity was plotted for different inhibitor (ZnCl_2_) concentrations for all the three OmpT samples.

### 2.5. OmpT Protease Activity Assay

Based on a method previously described by Kramer et al. (2000) [19], wild type and mutant OmpT samples were assayed for their protease activity. All fluorescence assays were performed in 384-well plates at a final reaction volume of 100 μL. The synthetic peptide substrate Abz-ARRA-Tyr (NO_2_)-NH_2_ was prepared as stock solutions of 0.5 mM in Milli-Q water and diluted with a mixture of Milli-Q water and 10× assay buffer (200 mM Bis-tris, 20 mM DodMe_2_NPrSO_3_, 50 mM EDTA, pH 6.7) to a final peptide concentration of 50 μM in 1× assay buffer (20 mM Bis-tris, 2 mM DodMe_2_NPrSO_3_, 5 mM EDTA, pH 6.7) for the assay. The concentration of the wild type and mutant OmpT samples were confirmed by a BCA assay. A stock solution of 2 mg/mL of LPS from *E. coli* was prepared in water. All four OmpT samples were diluted 80 times in a buffer devoid of LPS. The diluted OmpT samples were incubated with 20 µg/mL LPS for ~15 min, to ensure OmpT activity. An amount of 10 μL of the OmpT-LPS mixture was added to 90 μL of 50 μM peptide substrate in assay buffer. The enzymatic reaction was performed at 37 °C. The increase in fluorescence intensity upon OmpT addition was collected over time using a TECAN infinite^®^ M200 PRO (Männedorf, Switzerland) fluorescence spectrophotometer fitted with a plate reader. Fluorescence intensity signals were collected every 10 s for 60 min with the excitation wavelength set to 325 nm and emission at 420 nm, and the data plotted in OriginPro 9.1. Specific activity was calculated for each OmpT sample based on the fluorescence data.

### 2.6. Liquid Chromatography-Mass Spectrometry (LC-MS) and MALDI-TOF

OmpT samples were prepared for LC-MS analysis of soluble (9 kDa peptide) and detergent-stabilized (33.5 and 24.5 kDa proteins) species. The frozen OmpT sample was thawed on ice. The 9 kDa peptide was observed by dilution of the thawed sample in water to a final concentration of ~10 µM for injection to the LC-MS system. The two higher molecular weight species were prepared for LC-MS analysis using methanol/chloroform/water precipitation to remove the detergent [25]. The sample was resuspended in 5% acetonitrile and 0.1% formic acid in water to a concentration of 30 µM and diluted with water to a final concentration of ~1 µM. LC-MS analysis was performed on an Agilent 1200 series HPLC connected to an Agilent 6210A time-of-flight (TOF) mass spectrometer. A 10 µL injection of each sample was captured on a C18 trap column (Waters) and eluted using a gradient from 5% to 95% acetonitrile and 0.1% formic acid in water with a flowrate of 0.25 mL/min. Data were analyzed with Agilent MassHunter Qualitative Analysis B.04.00.

For the MALDI-TOF analysis, two synthetic peptides, Ac-GARKVG-NH2 and Ac-GARIIG-NH2, with and without a dibasic site, respectively, were employed to confirm the substrate specificity of the RC-31 (LPS) samples. A stock solution of 0.5 mM for each peptide was prepared and was stored at −20 °C. Each peptide was diluted using a mixture of Milli-Q water and 10× assay buffer (200 mM Bis-tris, 20 mM DodMe_2_NPrSO_3_, 50 mM EDTA, pH 6.7) to a final peptide concentration of 10 µM in 1× assay buffer (20 mM Bis-tris, 2 mM DodMe_2_NPrSO_3_, 50 mM EDTA, pH 6.7), to which OmpT was added at 1 µg/mL. The reaction mixture was incubated at 37 °C for 1 h with shaking at 500 rpm. HiPPR™ Detergent Removal Spin Column was used, as directed in the product guide, to remove the detergent from the reaction mixture. The reaction samples for the two peptides and the matrix, α-Cyano-4-hydroxycinnamic acid, were mixed (1:1), and 1 μL of the mixture was spotted on a MALDI-TOF array plate. The matrix and reaction sample was allowed to dry before loading into the instrument. The samples were analyzed using a Shimadzu Biotech Axima Performance Mass spectrometer (Nakagyo-ku, Kyoto, Japan) that contained a nitrogen UV laser. The intensity vs. m/z spectrum was collected at a laser power of 100 mV in the reflection hi-resolution mode, and other settings were adjusted for the best signal-to-noise ratio.

## 3. Results and Discussion

### 3.1. Refolding and Heat-Modifiable Migration of OmpT

**Figure 1:**
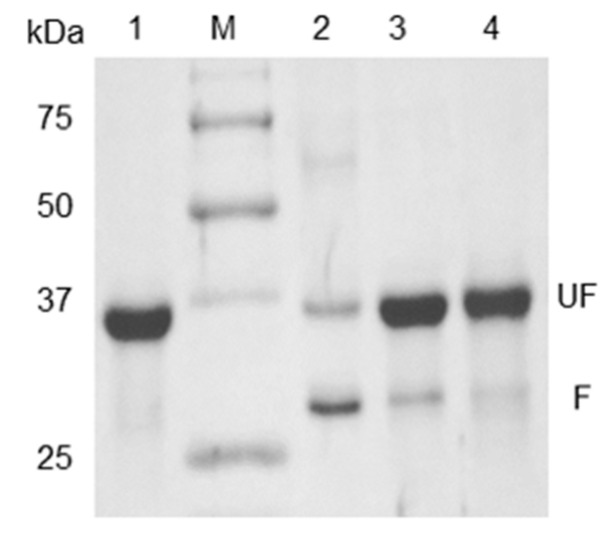
Gel-shifting of _mt_OmpT_DB_ on SDS-PAGE: Lane 1—_mt_OmpT_DB_ inclusion bodies (IBs), Lane M—protein molecular weight marker. _mt_OmpT_DB_ after refolding in DodMe_2_NPrSO_3_ detergent buffer when heated at 100 °C for 0 min (Lane 2), 10 min (Lane 3), and 45 min (Lane 4). UF—unfolded protein, F—folded protein.

A mutant OmpT (_mt_OmpT_DB_), with its dibasic site K_217_-R_218_ mutated to K_216_-G_217_-R_218_, was expressed in vitro as IBs. High yields and ease of separation from unwanted soluble proteins is an important advantage of expressing the protein as IBs in *E. coli* [19]. However, IBs are partially folded or misfolded insoluble protein aggregates that need to be solubilized and completely unfolded before properly refolding them back into their native state [26]. Therefore, _mt_OmpT_DB_ IBs were solubilized and unfolded in 8 M urea before they were refolded. The unfolding was confirmed by analyzing the sample on an SDS gel where the protein migrated to a distinct 33.5 kDa band (Figure 1, Lane 1). _mt_OmpT_DB_ IBs solubilized and unfolded in 8 M urea were subsequently refolded in the presence of 31 mM DodMe_2_NPrSO_3_ detergent. The criteria for selecting DodMe_2_NPrSO_3_ as the refolding detergent was based on previous studies by Kramer et al. (2000) [19] who screened Triton X-100, Tween 20, octylpolyoxyethylene (OPOE), and n-hexa-decylphosphorylcholine as alternatives to DodMe_2_NPrSO_3_. They did not observe any protease activity in these alternate detergents despite these detergents appearing to help properly refold the OmpT.

Unlike most proteins, OmpT and other Gram-negative outer membrane beta-barrel proteins are resistant to denaturation by SDS alone due to the extensive hydrogen bonding between the β-strands [27,28,29,30]. As a result, OmpTs treated with SDS alone continue to retain a compact globular shape allowing it to migrate further along the SDS-PAGE gel and be mistaken for a lower molecular weight protein of 27 kDa (Figure 1, Lane 2, lower MW band). This anomalous migration is termed “gel-shifting”, as the migration does not correlate with molecular weight. For a detailed description and discussion of the migration pattern of folded and unfolded OmpT see Figure S1. Therefore, in order for OmpT to migrate at its expected molecular weight of 33.5 kDa, a combination of SDS and heat treatment is required to unfold a folded OmpT (Figure 1, Lane 4). Additionally, it is important to note that in previously reported work [19] on the heat-modifiable migration of OmpT, unfolding was achieved within only 5 min of heat denaturation at 100 °C. However, we still observed a faint lower molecular weight band at 27 kDa (Figure 1, lane 3) even after 10 min of heating at 100 °C that disappeared only upon prolonged heating at 100 °C for 45 min (Figure 1, lane 4). Nevertheless, this heat-modifiable migration serves as a reliable indicator of OmpT refolding efficiency since it allows for the determination of the relative amounts of folded and unfolded protein in a given OmpT sample [19,31,32].

### 3.2. Autoproteolysis of OmpT

**Figure 2:**
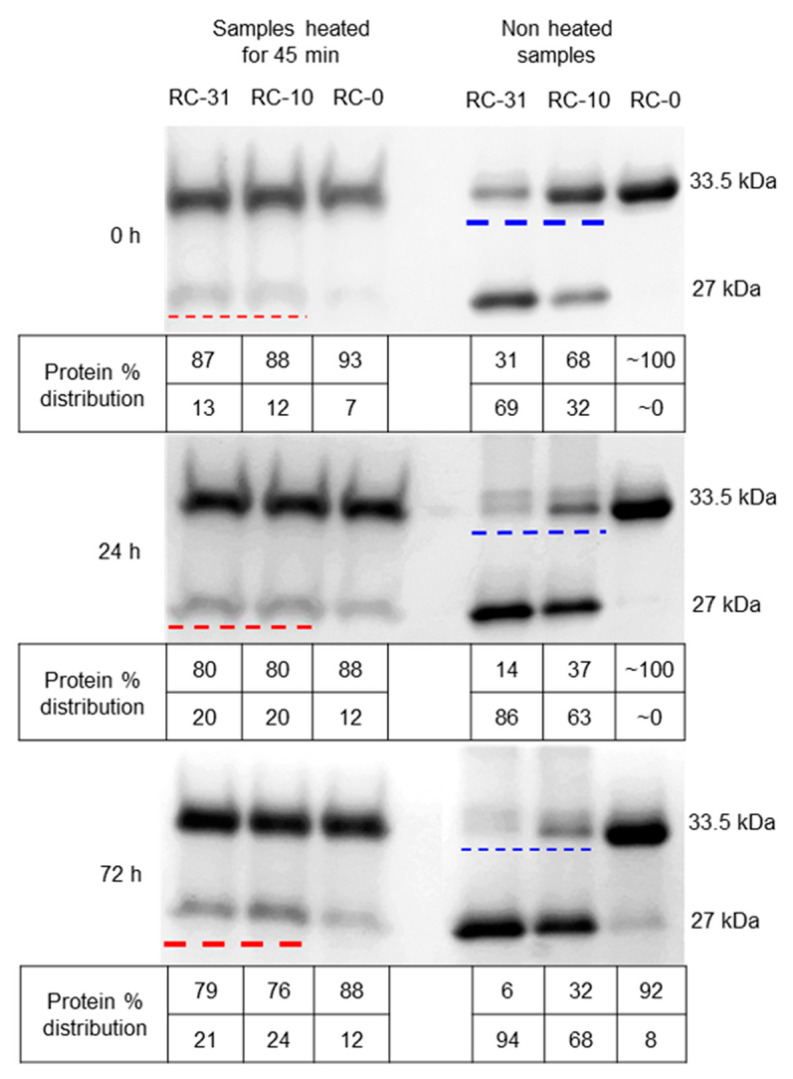
Solubilized OmpT IBs reconstituted in detergent micelles were monitored for autoproteolysis over a time period of 72 h: Solubilized OmpT IBs were refolded in the presence of 31, 10, or 0 mM DodMe_2_NPrSO_3_ detergent and denoted as RC-31, RC-10, and RC-0, respectively. Samples incubated at 4 °C were collected after 0, 24, and 72 h of refolding and analyzed using SDS-PAGE: Left panel—samples heated for 45 min prior to gel loading, and right panel—samples loaded without any heating (non-heated samples) on SDS-PAGE. The thickness of the red and blue dashed lines reflects increasing and decreasing protein band intensities. Note: We observed at least one additional higher molecular weight band (slightly above the 33.5 kDa) in the non-heated RC-31 and RC-10 samples of 24 and 72 h which were also observed by Kramer et al. (2000) [19]. These are most likely OmpT complexed with LPS.

In order to study OmpT autoproteolysis, wild type OmpT was expressed in vitro as IBs that were later solubilized and unfolded in 8 M urea. The unfolded proteins were subsequently refolded in Tris-HCl buffer containing 31, 10, or 0 mM DodMe_2_NPrSO_3_ detergent. For the sake of convenience, we refer to these three refolding conditions as RC-31, RC-10, and RC-0 denoting the high, intermediate, and zero detergent concentration, respectively. When OmpT IBs were refolded in the absence of the detergent (RC-0), the protein migrated as a single band at 33.5 kDa in an SDS-PAGE gel regardless of heat treatment, indicating that all of the OmpT was unfolded (Figure 2, non-heated and 45 min-heated samples, Lane RC-0). As previously reported, and confirmed here, the presence of the detergent during the refolding process aids in the proper refolding of OmpT from the IBs [33,34,35]. At this point, the apparent refolding efficiencies for RC-31 and RC-10 equal ~69% and 32%, respectively, based on the relative intensities of the two bands at 33.5 and 27 kDa for the non-heated samples (Figure 2, non-heated samples, Lane RC-31 and RC-10), a clear indication that excess detergent is essential to achieve higher refolding efficiencies. However, this is not the complete story as will be discussed below. The specific activity for the RC-31, RC-10, and RC-0 samples measured using a previously reported FRET assay [19] was calculated to be 13.01 nmol min^−1^ mg^−1^, 5.64 nmol min^−1^ mg^−1^, and close to 0 pmol s^−1^ (Figure S3—Day 0, data not shown for the RC-0 sample), and these values correlated well with the refolding efficiency (Figure 2, 0 h for the non-heated samples).

**Figure 3:**
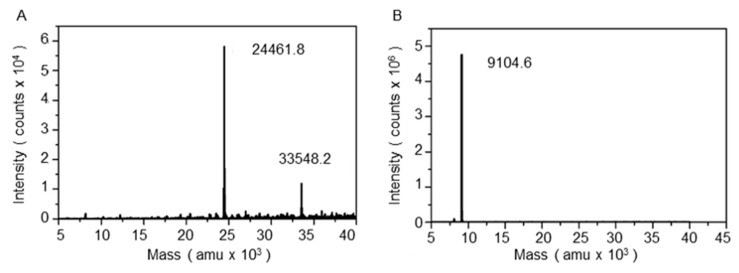
LC-MS characterization of the OmpT samples: (**A**) The larger autoproteolyzed fragment (24.4 kDa) and intact OmpT (33.5 kDa) after detergent removal via methanol/chloroform/water precipitation, and (**B**) the smaller proteolyzed fragment (9.1 kDa) from the soluble phase (i.e., without precipitation).

Despite the prolonged heat treatment for 45 min at 100 °C intended to unfold all OmpTs, both the RC-31 and RC-10 samples exhibited a faint band at 27 kDa (Figure 2, bands underlined with red dashed line for 0 h). This 27 kDa band is not due to folded OmpT, but in fact attributable to the larger of the two autoproteolytic fragments resulting from OmpT autoproteolysis (Scheme 1B). It is unfortunate, but the larger autoproteolytic fragment as well as the folded intact OmpT display an almost identical migration pattern through the gel with an apparent molecular weight of ~27 kDa [19], thus making data interpretation difficult and sometimes confusing. The LC-MS analysis of the samples analogous to RC-31 and RC-10 revealed peaks for the full-length protein at 33.5 and two peaks at 9.1 and 24.4 kDa for the autoproteolyzed fragments, thus confirming autoproteolysis as well as the site of autoproteolysis to be between K_217_ and R_218_ (Figure 3A,B; Supplementary Figure S2 and Supplementary Table S1). The RC-31 and RC-10 samples exhibited low levels (~12%) of autoproteolysis for the freshly refolded samples, based on the faint bands observed at 27 kDa (Figure 2, bands underlined with red dashed line for 0 h). The band intensities gradually increased to 20% and to 21–24% for the samples analyzed after incubation at 4 °C for 24 and 72 h, respectively (Figure 2, bands underlined with red dashed line for 24 h, and 72 h). During the same time frame, the non-heated RC-31 and RC-10 samples exhibited a gradual loss in intensity from 31% to 6% and 68% to 32%, respectively, of the 33.5 kDa band (Figure 2, bands underlined with blue dashed line for 0, 24, and 72 h). Autoproteolysis of unfolded OmpTs is the most likely explanation for this observation. If folded OmpTs were to undergo autoproteolysis, there would be a gradual decrease in protease activity for the RC-31 and RC-10 samples. However, the protease activity of the above RC-31 and RC-10 samples did not change significantly over time and retained ~80% activity even after 40 days when stored at 4 °C, suggesting that the folded OmpT did not undergo any extensive autoproteolysis (Supplementary Figure S3). Taken together, the gradual increase in the level of autoproteolytic fragment, a concomitant loss of the unfolded OmpT, and a constant protease activity of the folded OmpT sample over time clearly suggest that the unfolded portion of OmpT is prone to autoproteolysis.

Alternatively, it would be possible to follow OmpT autoproteolysis by monitoring the levels of the smaller (9.1 kDa) autoproteolyzed fragment using tricine-based gels. However, this smaller fragment carries a dibasic site in its sequence at K_259_-K_260_ which is prone to undergo further autoproteolysis yielding two fragments of ~4.9 and 4.2 kDa. Following autoproteolysis by monitoring the changing levels of three low molecular weight bands with two of them below 5 kDa is relatively difficult and is likely to be error prone when it comes to gathering quantitative data based on cumulative band intensities. In comparison, monitoring the ~27 kDa band, although challenging, is relatively less error prone due to its dependence on a single band intensity and more importantly, provides details of protein refolding efficiency.

**Figure 4:**
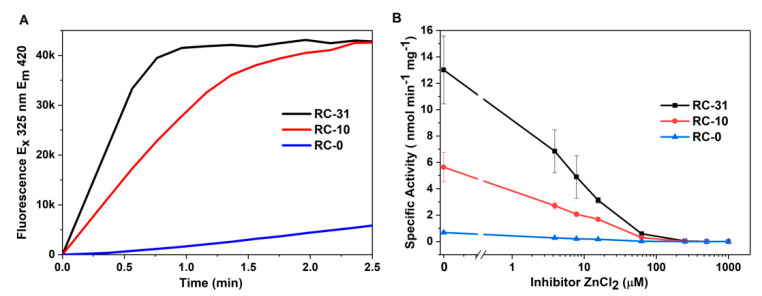
FRET cleavage and inhibitor assays for OmpT in three refolding buffers containing different concentrations of detergent, RC-31, RC-10, and RC-0. (**A**) Change in fluorescence emission intensity due to cleavage of FRET-peptide by OmpT. (B) Protease activity measurements in the presence of increasing inhibitor (ZnCl_2_) concentration and plotted as specific activity vs. inhibitor concentration. Note: To avoid the 0 value on the *X*-axis of the semi-logarithmic plot in (**B**), the specific activity corresponding to 0 µM ZnCl_2_ was plotted as 0.01 µM ZnCl_2_.

Interestingly, the RC-0 samples displayed little autoproteolysis despite having the highest levels of unfolded OmpT among the three refolding conditions (Figure 2, Lanes RC-0). This is likely due to the lack of sufficient amounts of properly refolded and active OmpT necessary to cleave other unfolded OmpTs. This was confirmed by conducting FRET-based protease activity measurements where RC-0 samples exhibited poor activity when compared with the RC-31 and RC-10 samples (Figure 4A, Blue). Furthermore, active site titration measurements using ZnCl_2_ as the inhibitor confirmed the RC-31 samples to contain a higher level of active OmpT as compared with RC-10, with the lowest values obtained for the RC-0 samples (Figure 4B). These activity observations correlated well with the refolding efficiencies of the three samples (Figure 2, non-heated samples, 0 h).

Although the initial refolding efficiency of ~69% and 32% for the RC-31 and RC-10 samples, respectively, were based on the relative intensities of the 33.5 and 27 kDa bands for the non-heated samples in Figure 2, this did not take into account the contribution of the autoproteolytic fragment to the 27 kDa band. The refolding efficiency was therefore recalculated as follows: *Refolding Eff (%) = [(% intensity of 27 kDa band for 0 h, non-heated samples) − (% intensity of 27 kDa band for 0 h, heated samples)]*. Basically, the contribution from the autoproteolyzed fragment is removed when calculating the refolding efficiency. For the RC-31 and RC-10 samples, the refolding efficiency was now close to ~60% and 20%, respectively. However, for the RC-31 samples, the refolding efficiency varied between 50–70% for different batches (data not shown) and was difficult to control using the protocol adopted from Kramer et al. (2000) [19]. Despite this difference in the protein refolding efficiency between the different batches, the gradual increase in the level of autoproteolysis and a concomitant loss of unfolded OmpT clearly suggest that the unfolded OmpT underwent autoproteolysis (Supplementary Figure S2).

### 3.3. Role of LPS in Autoproteolysis of Unfolded OmpT

LPS has previously been shown to be essential for OmpT’s activity [15,19,36,37,38,39]. Binding of LPS to a putative LPS-binding motif in OmpT is thought to cause a slight conformational change in the protease’s active site which is associated with an active OmpT [15,38]. Although no LPS was added during the refolding stage for RC-31, RC-10, and RC-0, autoproteolysis was still observed in all three samples, revealing that active OmpT was present during the refolding process. This could be due to the unavoidable residual LPS from the membrane fraction during the IB isolation process. Attempts to quantify this residual LPS were unsuccessful, mostly due to interference from detergent molecules. While LPS appears to be critical for autoproteolysis, it is also important to consider that not all of the OmpT is autoproteolyzed. Active OmpT, presumably with an LPS bound to it, seems to be resistant to autoproteolysis, suggesting a protective role for LPS. Similarly, Wu et al. (2012) [21] suspected a protective role of LPS against OmpT autoproteolysis and presented evidence to support this hypothesis by performing experiments where LPS was added to refolded OmpT immediately after cation exchange purification [21,22]. We therefore wondered if LPS is playing a dual role, that of activating OmpT and of protecting it from autoproteolysis.

**Figure 5:**
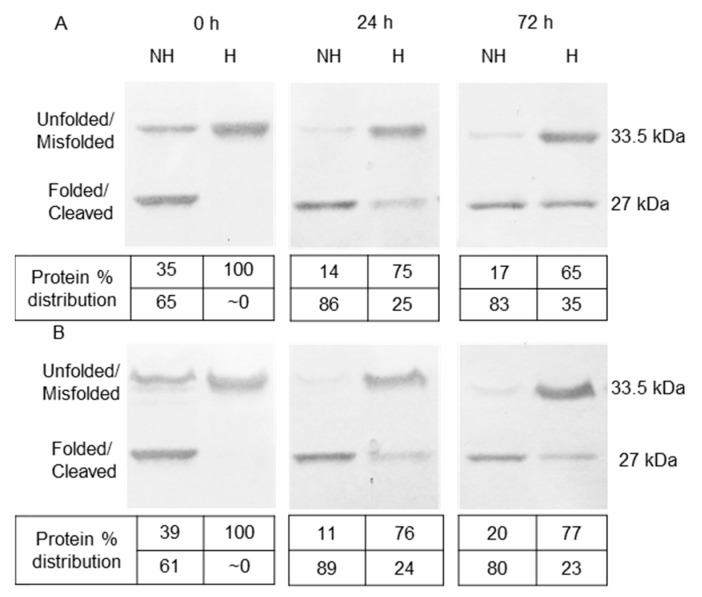
OmpT refolding in the presence and absence of LPS. (**A**) OmpT IBs refolded in absence of LPS. (**B**) OmpT IBs refolded in the presence of 4.5 mg/mL LPS. Samples were collected at 0, 24, and 72 h. Samples were either heated for 45 min at 100 °C (H) or not heated (NH) prior to gel loading.

To explore the role of LPS in OmpT autoproteolysis, OmpT IBs solubilized in 8 M urea were refolded in a buffer containing 31 mM detergent (RC-31) along with excess LPS (4.5 mg/mL) or no LPS. For the sake of convenience, we refer to these two refolding conditions as RC-31(LPS) and RC-31 to differentiate between samples refolded in the presence of excess or no externally added LPS, respectively. The RC-31(LPS) and RC-31 samples collected at three different intervals over a 72 h period and run on an SDS-PAGE exhibited an almost identical band intensity profile (Figure 5). The intensity of the 33.5 kDa bands corresponding to the unfolded protein decreased over time from 35% to 17% and 39% to 20% for RC-31(LPS) and RC-31, respectively (Figure 5A, Lanes NH (0, 72 h) and 5 B Lanes NH (0, 72 h)). During the same time, the 27 kDa protein band corresponding to the autoproteolytic fragment increased over time for both conditions from 0% to 35% and 0% to 23% for RC-31(LPS) and RC-31, respectively (Figure 5A, Lanes H (0, 72 h) and 6 B Lanes H (0, 72 h)). These almost identical autoproteolysis profiles for the RC-31(LPS) and RC-31 samples indicate that LPS did not protect the unfolded OmpT from autoproteolysis. The overall rate of autoproteolysis in the RC-31(LPS) and RC-31 samples during the observed time period were calculated to be 0.5%/h and 0.3%/h, indicating no significant difference between the two samples, suggesting that residual LPS is sufficient to keep OmpT in an active state. Furthermore, the overall refolding efficiency of the RC-31(LPS) and RC-31 samples analyzed immediately after refolding were calculated to be 65% and 61%, respectively, again indicating no significant difference in the refolding efficiency between the two samples. In summary, excess LPS did not affect autoproteolysis of the unfolded OmpT nor did it affect the refolding efficiency of OmpT IBs. Additionally, varying the levels of LPS during OmpT IB refolding did not significantly alter the above results (data not shown).

**Figure 6:**
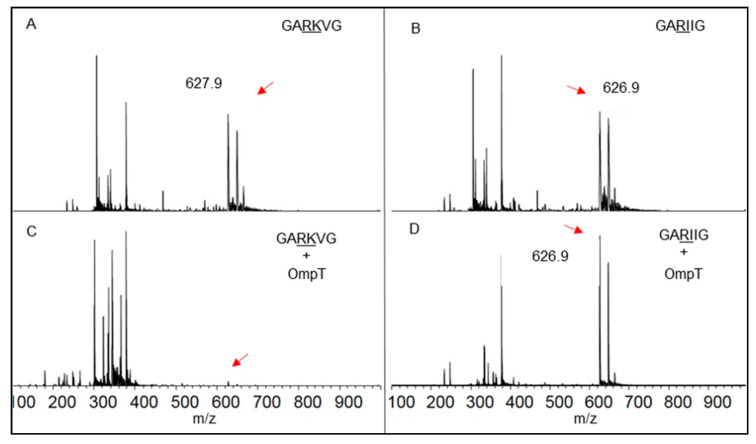
Characterization of substrate specificity of RC-31(LPS) for the dibasic sites: MALDI-TOF spectra of a short synthetic peptide with a dibasic site in the absence (**A**) and presence (**C**) of RC-31(LPS). MALDI-TOF spectra of a short synthetic peptide lacking a dibasic site in the absence (**B**) and presence (**D**) of RC-31(LPS). Red arrows point to the expected location of the m/z peaks for the uncleaved peptide.

MALDI-TOF experiments were undertaken to confirm that the substrate specificity of OmpT (RC-31(LPS) samples) for the dibasic sites was preserved (Figure 6), in agreement with the established substrate specificity reported in the literature [17,19,20]. When a short synthetic peptide (Seq: GARKVG, MW 627 Da; Figure 6A) carrying a dibasic site in its sequence was treated with OmpT, the signal for the full-length peptide was no longer visible in the MALDI spectra (Figure 6C), indicating complete cleavage of the peptide. A control peptide (Seq: GARIIG, MW 626 Da; Figure 6B) lacking a dibasic site in its sequence was not cleaved by OmpT, as seen from the peak arising from the full-length peptide (Figure 6D).

### 3.4. Mutation of the Predicted LPS Biding Residues in OmpT

From the above section, it is evident that LPS does not protect unfolded OmpT from autoproteolysis, but to understand whether LPS protects the folded OmpT from autoproteolysis, it is important to obtain folded OmpT without any LPS bound to it. This is easier said than done, since it is extremely difficult to separate and purify OmpT IBs without residual LPS from the membrane fraction during the isolation process. Chromatographic columns and kits designed for endotoxin removal were not considered due to the harsh conditions the protein must endure during the process [40]. Moreover, they are not known to completely remove LPS, especially those that are bound to the protein as co-factors. The other alternative is to express a mutant OmpT whose LPS binding site is modified such that it can no longer bind LPS. According to Vandeputte-Rutten et al. (2001) [1], one LPS molecule per OmpT is sufficient for OmpT activity. This one LPS is thought to bind to a putative LPS-binding motif in OmpT whose location was predicted based on the crystal structure of FhuA in complex with LPS [1]. FhuA is an outer membrane β-barrel protein in *E. coli* that shares some structural similarity with OmpT, and has a known LPS-binding motif [41]. In total, five residues of FhuA’s LPS-binding motif are also present in OmpT at almost the exact same positions, and these are: Arg_138_, Arg_175_, Lys_226_, Glu_136_, and Tyr_134_ on OmpT (Scheme 1) [1].

Two recombinant mutant OmpTs with either the three basic residues (_mt3_OmpT_LPS_) or all five (_mt5_OmpT_LPS_) of the predicted LPS binding residues modified were expressed and purified (Table 1). LPS should not bind to the modified putative LPS-binding motif in the two mutants and ought to remain inactive and exhibit no autoproteolysis.

**Figure 7:**
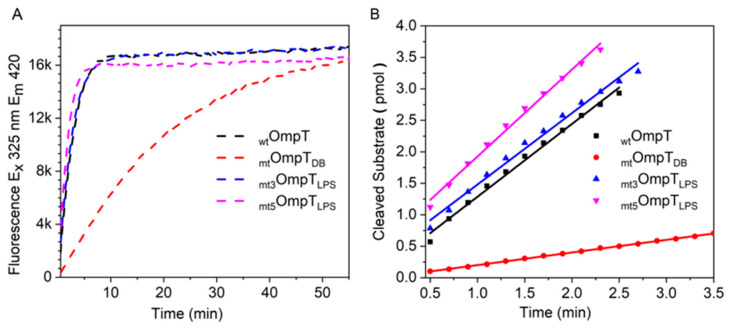
Comparison of enzyme activities of the four OmpT variants (OmpT, _mt_OmpT_DB_, _mt3_OmpT_LPS_, and _mt5_OmpT_LPS_): (**A**) FRET-based activity measurements; (**B**) initial velocity measurements used to calculate specific activity, Table 1.

Before we could discern the role of LPS in autoproteolysis, a FRET-based activity assay was performed to confirm that the two mutants were inactive. Surprisingly, _mt3_OmpT_LPS_ and _mt5_OmpT_LPS_ exhibited low levels of protease activity that significantly improved with the addition of LPS (Figure 7, Table 1 and Figure S4) indicating that the putative LPS-binding motif in OmpT is not critical for its activity. Nor can we confirm the role of LPS binding to the putative LPS-binding motif in protecting active OmpT from autoproteolysis. However, we cannot conclusively rule out the possibility that LPS still binds to OmpT and modifies its activity as well as protects it from autoproteolysis. It is therefore tempting to hypothesize that there may be other LPS binding sites on OmpT that are critical for its activity and capable of protecting it from autoproteolysis. In an attempt to independently determine the number of LPS molecules that bind to OmpT, as well as to its mutants, we performed isothermal titration calorimetry (ITC) experiments. Unfortunately, we were unable to get any clear results due to technical difficulties in distinguishing between LPS binding to OmpT and LPS binding to the detergent micelles in which the OmpT was reconstituted (data not shown). However, previous studies with OprH, an outer membrane protein similar to OmpT, report that LPS binds non-specifically to OprH and it is possible that this is also the case with OmpT [42,43]. Their observation is in line with our hypothesis and prompts for further in-depth studies to identify the LPS binding sites that are critical for OmpT’s activity and capable of protecting OmpT from autoproteolysis, if such sites do exist.

## 4. Conclusions

Based on the above findings, it is clear that only the unfolded OmpT undergoes autoproteolysis in vitro and LPS was unable to protect unfolded OmpT from undergoing autoproteolysis. A practical implication of this finding is that in vitro OmpT autoproteolysis can be reduced by improving protein refolding efficiencies such that there is less unfolded OmpT available for autoproteolysis. Folded active OmpT remains resistant to autoproteolysis since its protease activity did not significantly change over almost 40 days. However, we were unable to confirm if this protection from autoproteolysis was due to LPS. Additionally, the possible incorporation of LPS into the detergent micelles may alter the normal interaction of LPS with OmpT, thus influencing the level of autoproteolysis and activity. In this regard, we are currently investigating OmpT reconstitution in supported lipid bilayers composed of different ratios of phospholipid and LPS, and whether these different membrane compositions affect OmpT (to be reported elsewhere). Together with additional structural data focusing on the OmpT–LPS interaction, we should be able to gain better insights into the role and mechanism of LPS in activating OmpT in vitro and in vivo. Moreover, the previously proposed [15] putative LPS-binding motif in OmpT appears not to be critical for its activity since mutant OmpTs, with the predicted LPS-binding residues mutated, exhibited excellent protease activity. Thus, it is possible that there might be multiple LPS binding sites present on OmpT or that LPS binding to OmpT is purely non-specific [42,43]. Finally, the data presented herein reiterate the need to exercise caution when interpreting the SDS-PAGE profiles of heat-modifiable proteins where reliable results are obtained only upon prolonged heating of the samples.

## Figures and Tables

**Table 1:** Specific activity of wild type OmpT and the various mutants used in this study.

OmpT I.D.	Residues Mutated *	Specific Activity (pmol min^−1^ mg^−1^)
OmpT	None	1.3 × 10^4^
_mt_OmpT_DB_	G_216_K and K_217_G	1.6 × 10^3^
_mt3_OmpT_LPS_	R_138_E, R_175_E, K_226_E	1.5 × 10^4^
_mt5_OmpT_LPS_	Y_134_A, E_136_A, R_138_E, R_175_E, K_226_E	1.6 × 10^4^

* For example, Y_134_A means the phenylalanine at position 134 was mutated to an alanine.

## Supplementary Materials

The following are available online at https://www.mdpi.com/2218-273X/10/6/922/s1, Figure S1: Autoproteolysis of OmpT over time: OmpT samples were collected at 0, 24, and 72 h after refolding. The samples were run on SDS-PAGE: (A) Without heating the samples (B) Samples that were heated for 45 min before loading on SDS-PAGE. Figure S2: Mass spectrum of OmpT collected by LC-MS before deconvolution. Figure S3: Protease activity of RC-31 and RC-10 samples monitored over 40 days. OmpT IBs were refolded in presence of different concentrations of detergent and their kinetic activity was measured using the FRET peptide substrate (Abz-ARRA -Tyr (NO2)-NH2) over 40 days. Figure S4. OmpT protease activity in the presence and absence of externally added LPS. FRET based assay was used to measure protease activity of (A) wild type OmpT, (B) mtOmpTDB, (C) mt3OmpTLPS, (D) mt5OmpTLPS in the presence (red) and absence (blue) of LPS. Black lines indicate fluorescence intensity of control samples containing FRET peptide with LPS, but no OmpT added. Table S1: Molecular weight of OmpT and the fragments of OmpT.

## References

[1] Vandeputte-Rutten L., Kramer R.A., Kroon J., Dekker N., Egmond M.R., Gros P. (2001). Crystal structure of the outer membrane protease OmpT from *Escherichia coli* suggests a novel catalytic site. EMBO J..

[2] Hritonenko V., Stathopoulos C. (2007). Omptin proteins: An expanding family of outer membrane proteases in Gram-negative Enterobacteriaceae. Mol. Membr. Biol..

[3] Stumpe S., Schmid R., Stephens D.L., Georgiou G., Bakker E.P. (1998). Identification of OmpT as the protease that hydrolyzes the antimicrobial peptide protamine before it enters growing cells of *Escherichia coli*. J. Bacteriol..

[4] Brannon J.R., Thomassin J.-L., Desloges I., Gruenheid S., Le Moual H. (2013). Role of uropathogenic Escherichia coli OmpT in the resistance against human cathelicidin LL-37. FEMS Microbiol. Lett..

[5] Hui C.-Y., Guo Y., He Q.-S., Peng L., Wu S.-C., Cao H., Huang S.-H. (2010). Escherichia coli outer membrane protease OmpT confers resistance to urinary cationic peptides. Microbiol. Immunol..

[6] Nielubowicz G.R., Mobley H.L.T. (2010). Host-pathogen interactions in urinary tract infection. Nat. Rev. Urol..

[7] Flores-Mireles A.L., Walker J.N., Caparon M., Hultgren S.J. (2015). Urinary tract infections: Epidemiology, mechanisms of infection and treatment options. Nat. Rev. Microbiol..

[8] Thomassin J.-L., Brannon J.R., Gibbs B.F., Gruenheid S., Le Moual H. (2012). OmpT outer membrane proteases of enterohemorrhagic and enteropathogenic *Escherichia coli* contribute differently to the degradation of human LL-37. Infect. Immun..

[9] Montero D., Orellana P., Gutiérrez D., Araya D., Salazar J.C., Prado V., Oñate Á., Canto F.D., Vidal R. (2014). Immunoproteomic Analysis To Identify Shiga Toxin-Producing *Escherichia coli* Outer Membrane Proteins Expressed during Human Infection. Infect Immun..

[10] Hartland E.L., Leong J.M. (2013). Enteropathogenic and enterohemorrhagic *E. Coli*: Ecology, pathogenesis, and evolution. Front. Cell. Infect. Microbiol..

[11] Thomassin J.L., Brannon J.R., Kaiser J., Gruenheid S., Le Moual H. (2012). Enterohemorrhagic and enteropathogenic *Escherichia coli* evolved different strategies to resist antimicrobial peptides. Gut Microbes..

[12] Urashima A., Sanou A., Yen H., Tobe T. (2017). Enterohaemorrhagic *Escherichia coli* produces outer membrane vesicles as an active defence system against antimicrobial peptide LL-37. Cell. Microbiol..

[13] Premjani V., Tilley D., Gruenheid S., Le Moual H., Samis J.A. (2014). Enterohemorrhagic *Escherichia coli* OmpT regulates outer membrane vesicle biogenesis. FEMS Microbiol. Lett..

[14] Kramer R.A., Vandeputte-Rutten L., De Roon G.J., Gros P., Dekker N., Egmond M.R. (2001). Identification of essential acidic residues of outer membrane protease OmpT supports a novel active site. FEBS Lett..

[15] Kramer R.A., Brandenburg K., Vandeputte-Rutten L., Werkhoven M., Gros P., Dekker N., Egmond M.R. (2002). Lipopolysaccharide regions involved in the activation of *Escherichia coli* outer membrane protease OmpT. Eur. J. Biochem..

[16] Sugimura K., Nishihara T. (1988). Purification, characterization, and primary structure of *Escherichia coli* protease VII with specificity for paired basic residues: Identity of protease VII and OmpT. J. Bacteriol..

[17] Dekker N., Cox R.C., Kramer R.A., Egmond M.R. (2001). Substrate Specificity of the Integral Membrane Protease OmpT Determined by Spatially Addressed Peptide Libraries. Biochemistry..

[18] Rollauer S.E., Sooreshjani M.A., Noinaj N., Buchanan S.K. (2015). Outer membrane protein biogenesis in Gram-negative bacteria. Philos. Trans. R. Soc. B Biol. Sci..

[19] Kramer R.A., Zandwijken D., Egmond M.R., Dekker N. (2000). In vitro folding, purification and characterization of *Escherichia coli* outer membrane protease OmpT. Eur. J. Biochem..

[20] Wood S.E., Sinsinbar G., Gudlur S., Nallani M., Huang C.-F.F., Liedberg B., Mrksich M. (2017). A Bottom-Up Proteomic Approach to Identify Substrate Specificity of Outer-Membrane Protease OmpT. Angew. Chemie Int. Ed..

[21] Wu C., Tran J.C., Zamdborg L., Durbin K.R., Li M., Ahlf D.R., Early B.P., Thomas P.M., Sweedler J.V., Kelleher N.L. (2012). A protease for “middle-down” proteomics. Nat. Methods..

[22] WU C. (2013). Development and Application of Ompt Based Middle down Proteomics. Ph.D. Thesis.

[23] Kudva R., Denks K., Kuhn P., Vogt A., Müller M., Koch H.-G. (2013). Protein translocation across the inner membrane of Gram-negative bacteria: The Sec and Tat dependent protein transport pathways. Res. Microbiol..

[24] Hirel P.H., Schmitter M.J., Dessen P., Fayat G., Blanquet S. (1989). Extent of N-terminal methionine excision from *Escherichia coli* proteins is governed by the side-chain length of the penultimate amino acid. Proc. Natl. Acad. Sci. USA.

[25] Wessel D., Flügge U.I. (1984). A method for the quantitative recovery of protein in dilute solution in the presence of detergents and lipids. Anal. Biochem..

[26] Carrió M.M., Villaverde A. (2005). Localization of chaperones DnaK and GroEL in bacterial inclusion bodies. J. Bacteriol..

[27] Noinaj N., Kuszak A.J., Buchanan S.K. (2015). Heat Modifiability of Outer Membrane Proteins from Gram-Negative Bacteria. Methods Mol. Biol..

[28] Koebnik R. (1999). Structural and Functional Roles of the Surface-Exposed Loops of the β-Barrel Membrane Protein OmpA from *Escherichia coli*. J. Bacteriol..

[29] Sánchez S., Arenas J., Abel A., Criado M.-T., Ferreirós C.M. (2005). Analysis of Outer Membrane Protein Complexes and Heat-Modifiable Proteins in Neisseria Strains Using Two-Dimensional Diagonal Electrophoresis. J. Proteome Res..

[30] Minetti C.A., Tai J.Y., Blake M.S., Pullen J.K., Liang S.M., Remeta D.P. (1997). Structural and functional characterization of a recombinant PorB class 2 protein from Neisseria meningitidis. Conformational stability and porin activity. J. Biol. Chem..

[31] Visudtiphole V., Thomas M.B., Chalton D.A., Lakey J.H. (2005). Refolding of *Escherichia coli* outer membrane protein F in detergent creates LPS-free trimers and asymmetric dimers. Biochem. J..

[32] Hong H., Tamm L.K. (2004). Elastic coupling of integral membrane protein stability to lipid bilayer forces. Proc. Natl. Acad. Sci. USA.

[33] Tulumello D.V., Deber C.M. (2012). Efficiency of detergents at maintaining membrane protein structures in their biologically relevant forms. Biochim. Biophys. Acta Biomembr..

[34] Anandan A., Vrielink A. (2016). Detergents in membrane protein purification and crystallisation. Adv. Exp. Med. Biol..

[35] Kleinschmidt J.H., Wiener M.C., Tamm L.K. (1999). Outer membrane protein A of *E. coli* folds into detergent micelles, but not in the presence of monomeric detergent. Protein Sci..

[36] Brandenburg K., Garidel P., Schromm A.B., Andrae J., Kramer A., Egmond M., Wiese A., Andra J., Kramer A., Egmond M. (2005). Investigation into the interaction of the bacterial protease OmpT with outer membrane lipids and biological activity of OmpT:lipopolysaccharide complexes. Eur. Biophys. J..

[37] Ebbensgaard A., Mordhorst H., Aarestrup F.M., Hansen E.B. (2018). The Role of Outer Membrane Proteins and Lipopolysaccharides for the Sensitivity of *Escherichia coli* to Antimicrobial Peptides. Front. Microbiol..

[38] Eren E., Van Den Berg B. (2012). Structural basis for activation of an integral membrane protease by lipopolysaccharide. J. Biol. Chem..

[39] Kukkonen M., Suomalainen M., Kyllönen P., Lähteenmäki K., Lång H., Virkola R., Helander I.M., Holst O., Korhonen T.K., Kyllonen P. (2004). Lack of O-antigen is essential for plasminogen activation by Yersinia pestis and Salmonella enterica. Mol. Microbiol..

[40] Ongkudon C.M., Chew J.H., Liu B., Danquah M.K. (2012). Chromatographic Removal of Endotoxins: A Bioprocess Engineer’s Perspective. ISRN Chromatogr..

[41] Ferguson A.D., Welte W., Hofmann E., Lindner B., Holst O., Coulton J.W., Diederichs K. (2000). A conserved structural motif for lipopolysaccharide recognition by procaryotic and eucaryotic proteins. Structure.

[42] Kucharska I., Liang B., Ursini N., Tamm L.K. (2016). Molecular Interactions of Lipopolysaccharide with an Outer Membrane Protein from Pseudomonas aeruginosa Probed by Solution NMR. Biochemistry.

[43] Edrington T.C., Kintz E., Goldberg J.B., Tamm L.K. (2011). Structural basis for the interaction of lipopolysaccharide with outer membrane protein H (OprH) from Pseudomonas aeruginosa. J. Biol. Chem..

