# Supplementary material for: Role of Lipopolysaccharide in Protecting OmpT from Autoproteolysis during In Vitro Refolding
Source: Biomolecules. 2020 Jun 18;10(6):922. doi: 10.3390/biom10060922 (PMC7356225; doi:10.3390/biom10060922)
Supplement: Supplementary file 1 [file biomolecules-10-00922-s001.pdf]

Electronic Supplementary Information

Article

Role of lipopolysaccharide in protecting OmpT from autoproteolysis during *in vitro* refolding

Gaurav Sinsinbar[1‡], Sushanth Gudlur[1‡], Kevin J Metcalf[2], Milan Mrksich[2], Madhavan Nallani[1], and Bo Liedberg[1*]

[1] Center for Biomimetic Sensor Science, School of Materials Science and Engineering, Nanyang Technological University, Singapore 637553; gaurav011@e.ntu.edu.sg (G.S.); sgudlur@ntu.edu.sg (S.G.); MNallani@ntu.edu.sg (M.N.)

[2] Departments of Chemistry and Biomedical Engineering, Northwestern University, Evanston, IL 60208, USA. ; kevin.metcalf@northwestern.edu (K.J.M.); milan.mrksich@northwestern.edu (M.M.)

‡ G.S and S.G contributed equally to this work

* Correspondence: bliedberg@ntu.edu.sg;

LPS

Material: According to the manufacturer (Sigma Aldrich, now MERCK) from where the LPS was purchased, the LPS is from *E. coli* O111:B4 and was purified by phenol extraction. The purchased LPS was not altered in anyway. The manufacturer's recommended protocol was followed for preparing the aqueous solution with the necessary warming to 70-80 ºC before use. Based on the information provided on the manufacturer's website, the LPS is most likely a heterogeneous mixture of the complete LPS with its O-antigen, core polysaccharides, and lipid A tail.

Gel-shifting and autoproteolysis of OmpT

Methods

OmpT IBs as pellets were solubilized in a solubilizing buffer (20 mM Tris-HCl, 8 M urea, 50 mM glycine, pH 8.3) and kept on ice for 1 h before the addition of pre-chilled refolding buffer (20 mM Tris-HCl, 31 mM $DodMe_2NPrSO_3$ pH 7.5) such that the final concentration of protein and urea was between 0.8-1 mg/mL and 0.8 M respectively. OmpT samples after refolding were incubated at 4 °C and were collected at different time intervals and run on SDS-PAGE gel after no (Figure S1 A) and 45 min (Figure S1 B) of heating at 100 °C.

Results

Bacterial outer membrane proteins like OmpT, OmpA, OmpF etc. referred to as 'heat-modifiable proteins', exhibit anomalous SDS-PAGE migration, in that heat treated samples will migrate differently than samples that have not been heated. This anomalous migration is termed 'gel-shifting' as the migration does not correlate with formula molecular weight. The reason for such anomalous migration on an SDS-gel is due to the protein's β-barrel structure, a robust tertiary structure consisting of an extensive hydrogen bonding network that hold the beta strands together. This structural feature makes it resistant towards heat denaturation, protease digestion, and chemical denaturation with urea and GdnHCl. As a result, they are not denatured by SDS alone and will retain their compact shape (while also binding less SDS) and hence migrate further along the gel [1–3]. Only upon heating do the proteins completely unfold and migrate to

its correct molecular weight. The gel-shifting property of outer membrane proteins provides an attractive route for determining tertiary structure formation of OmpA and other outer membrane proteins [4].

A folded heat-modifiable $_{mt}$OmpT$_{DB}$ when treated with SDS alone exhibits gel-shifting and migrates to an apparent molecular weight of 27 kDa (data not available) and when this $_{mt}$OmpT$_{DB}$ is treated with SDS and heated at 100 °C, it unfolds completely and migrates to its correct formula weight of 33.5 kDa (Figure 1, lane 4). Thus, a sample containing a mixture of folded and unfolded $_{mt}$OmpT$_{DB}$ when treated with SDS-alone exhibits two bands, at 27 kDa and 33.5 kDa (Figure 1, lane 2) corresponding to the folded and unfolded OmpT, respectively. Upon heating the above sample, the 27 kDa band (Figure 1, lane 3 and 4) disappears, thus confirming that it was indeed the folded $_{mt}$OmpT$_{DB}$. This method of analysis is commonly used to determine refolding efficiency of β-barrel proteins [5–7] and is how the refolding efficiency of OmpT is determined in the current study.

Unfortunately, OmpT is known to undergo autoproteolysis, and the larger autoproteolytic fragment migrates with an apparent molecular weight of around 27 kDa, which happens to be the same region for a folded OmpT. The autoproteolytic fragment does not exhibit gel-shifting [5] and will always migrate as a 27 kDa band regardless of heat treatment. Hence, an easy way to differentiate the autoproteolytic fragment from a folded OmpT is to simply heat the sample. If the 27 kDa band persists despite the heat treatment, then it belongs to the larger autoproteolytic fragment (Figure S1 B, 27 kDa band).

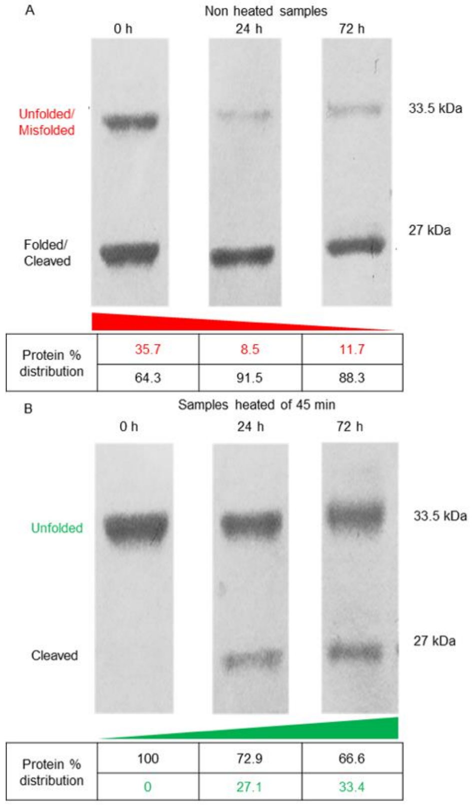

Figure S1: Autoproteolysis of OmpT over time: OmpT samples were collected at 0, 24, and 72 h after refolding. The samples were run on SDS-PAGE: (A) Without heating the samples (B) Samples that were heated for 45 min before loading on SDS-PAGE.

OmpT samples that were refolded from IBs and stored in 4 °C were collected at different time intervals and run on SDS-PAGE gel after no (Figure S1 A) and 45 min (Figure S1 B) of heating. Based on the ratio of the folded to unfolded OmpT bands, the refolding efficiency was close to 65 % (*Refolding Eff (%) = [(% intensity of 27 kDa band for 0 h, non-heated samples) – (% intensity of 27 kDa band for 0 h, heated samples)])* (Figure S1 A

& B 0 h). However, it is interesting to note that the high molecular weight band corresponding to the unfolded OmpT began to disappear with time and was reduced from ~36 % to ~12 % (Figure S1 A, Lanes – 0, 24, and 72 h.). This could indicate one of two things: either the unfolded OmpT is slowly refolding into its tertiary structure or the unfolded OmpT is undergoing autoproteolysis. To determine which of the two possibilities is true, the experiment was repeated, but this time the samples were heated to completely unfolded OmpT before running them on a gel. SDS-PAGE data shows that completely unfolded OmpT (Figure S1 B, Lane 0 h) immediately after it was refolded into detergent micelles exhibited no band visible at 27 kDa. However, with time, the 27 kDa band began to slowly increase in intensity from 0 to ~33% (Figure S1 B, Lanes 0, 24 and 72 h) in a manner similar to the disappearance of the high molecular weight band at 33.5 kDa for unfolded OmpT described above (Fig. S1A Lanes 0, 24 and 72 h). Taken together, this disappearance of the 33.5 kDa band and a concomitant appearance of a 27 kDa band strongly suggests that it is the unfolded OmpT that undergoes autoproteolysis.

LC-MS characterisation of refolded OmpT

Methods

OmpT samples were prepared for LC-MS analysis of a soluble specie (9 kDa peptide) and detergent-stabilized species (33.5 and 24.5 kDa proteins). Frozen OmpT sample was thawed on ice. The 9 kDa peptide was observed by dilution of the thawed sample in water to a final concentration of ~10 μM for injection to LC-MS system. The two higher molecular weight species were prepared for LC-MS analysis using methanol/chloroform/water precipitation to remove detergent [8]. Sample was resuspended in 5 % acetonitrile and 0.1 % formic acid (Thermo) in water to a concentration of 30 μM and diluted with water to a final concentration of ~1 μM. LC-MS analysis was performed on an Agilent 1200 series HPLC connected to an Agilent 6210A time-of-flight (TOF) mass spectrometer. A 10 μL injection of each sample was captured on a C18 trap column (Waters) and eluted using a gradient from 5 % to 95 % acetonitrile and 0.1 % formic acid in water with a flowrate of 0.25 mL/min. Data was analyzed with Agilent MassHunter Qualitative Analysis B.04.00.

Results

LC-MS analysis of refolded OmpT samples before deconvolution is shown below (Figure S2). Mass spectrum of OmpT sample precipitated with methanol/chloroform/water precipitation protocol shows multiply charged ion spectrum consistent with full-length OmpT and peptide 1 (Figure S2 A). The sample showed good S/N for the two higher MW species, full length (Figure 3 A – 33548.2) and peptide 1 (Figure 3 A – 24461.8). Mass spectrum of OmpT in Figure S2 A (peaks >~800 m/z) is consistent with these two expected species. Deconvolution by maximum entropy shows these two species (full length and peptide 1) as the most prominent peaks in Figure 3 A. Mass spectrum of OmpT sample from the soluble phase (i.e., without precipitation) shows multiply charged ion spectrum consistent with peptide 2 (i.e., low MW peptide 9,104.62). The mass spectrum in Figure S2 B (peaks >~800 m/z) display few peaks, but their spectrum is consistent with the lower MW species (peptide 2). Deconvolution by maximum entropy shows this species as the most prominent peak in Figure 3 B, albeit with low S/N. The mass of the peaks is presented in Table S1, with a comparison to the expected mass. Thus, the 33.5 kDa (Figure S2 A) and two peaks at 9.1 kDa and 24.4 kDa (Figure S2 A & B) for the autoproteolyzed fragments confirming autoproteolysis as well as the site of autoproteolysis, which was between K217-R218 (Table S1).

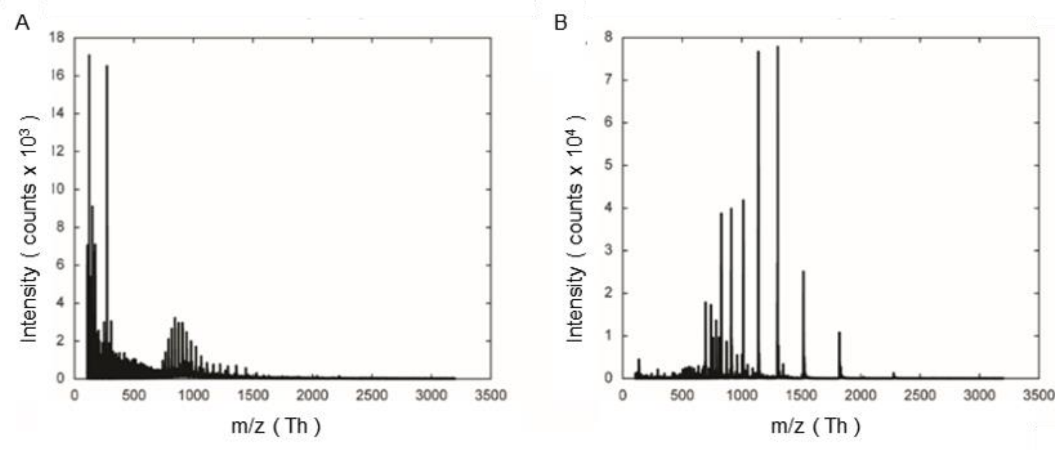

Figure S2: Mass spectrum of OmpT collected by LC-MS before deconvolution.

Table S1 : Molecular weight of OmpT and its autoproteolytic fragments.

OmpT Species	Calculated Mass (From aa sequence)	LC-MS	
		MCW[1] precipitation	TCA[2] precipitation
**Full length (298 aa)**	33548.79	33,548.18	n.d.
**Peptide 1 (218 aa)**	24462.81	24,461.77	n.d.
**Peptide 2 (80 aa)**	9,103.99	n.d.	9,104.62

1 Methanol:Chloroform:Water
2 Trichloroacetic Acid

Long term stability of OmpT activity

Method

OmpT was refolded as described in section 2.3 under two refolding conditions - RC-31 and RC-10 (described in section 3.1). Based on a method previously described by Kramer et al.[5], OmpT refolded under RC-31 and RC-10 condition were assayed for their protease activity.

Result

The proteolytic activity of RC-31 and RC-10 samples measured using a previously reported FRET assay [5] was calculated to be 10.2 pmol sec^{-1} and 5.5 pmol sec^{-1}, Figure S3- Day 0.

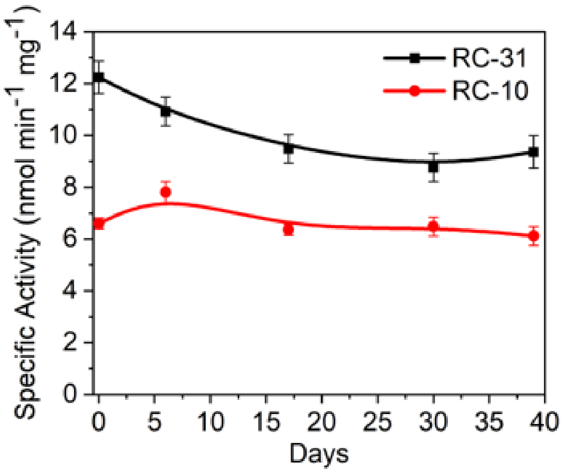

Figure S3: Protease activity of RC-31 and RC-10 samples monitored over 40 days. OmpT IBs were refolded in presence of different concentrations of detergent and their kinetic activity was measured using the FRET peptide substrate (Abz-ARRA -Tyr (NO2)-NH2) over 40 days.

OmpT activity in the presence and absence of externally added LPS

Method

Based on a FRET-based method previously described by Kramer et al. [5], wild type and mutant OmpT samples were assayed for their protease activity. All fluorescence assays were performed in 384 well plates at a final reaction volume of 100 µL. The synthetic peptide substrate, Abz-ARRA-Tyr (NO_2)-NH_2 was prepared as stock solutions of 0.5 mM in Milli-Q water and diluted with mixture of Milli-Q water and 10× assay buffer (200 mM Bis-tris, 20 mM $DodMe_2NPrSO_3$, 50 mM EDTA, pH 6.7) to a final peptide concentration of 10 µM in 1× assay buffer (20 mM Bis-tris, 2 mM $DodMe_2NPrSO_3$, 50 mM EDTA, pH 6.7), for the assay. A stock solution of 2 mg/mL of LPS from *E. coli* was prepared in water. All four OmpT samples were diluted either 200 or 500 times in a buffer devoid of LPS. The diluted OmpT samples were incubated with 100 µg/mL LPS warmed to 70°C. 10 µL of the OmpT-LPS mixture was added to 90 µL of 10 µM peptide substrate in assay buffer. The enzymatic reaction was performed at 37 °C. The increase in fluorescence intensity upon OmpT addition was collected over time using a TECAN infinite® M200 PRO (Männedorf, Switzerland) fluorescence spectrophotometer fitted with a plate reader. Fluorescence intensity signals were collected every 15 s for 57 min with the excitation wavelength set to 325 nm and emission at 420 nm, and the raw fluorescence intensity data was plotted in OriginPro 9.1 (OriginLab Corporation, Northampton, MA, USA).

Result

Wild type recombinant OmpT and all three of its mutants exhibited low levels of protease activity that significantly improved with the addition of LPS (see Figure S4 below). The protease activity of samples lacking LPS (Figure S4 Blue line) varied between wild type OmpT and its three mutants. This variation in activity is likely due to different amounts of residual LPS in the samples rather than an effect of the mutation itself.

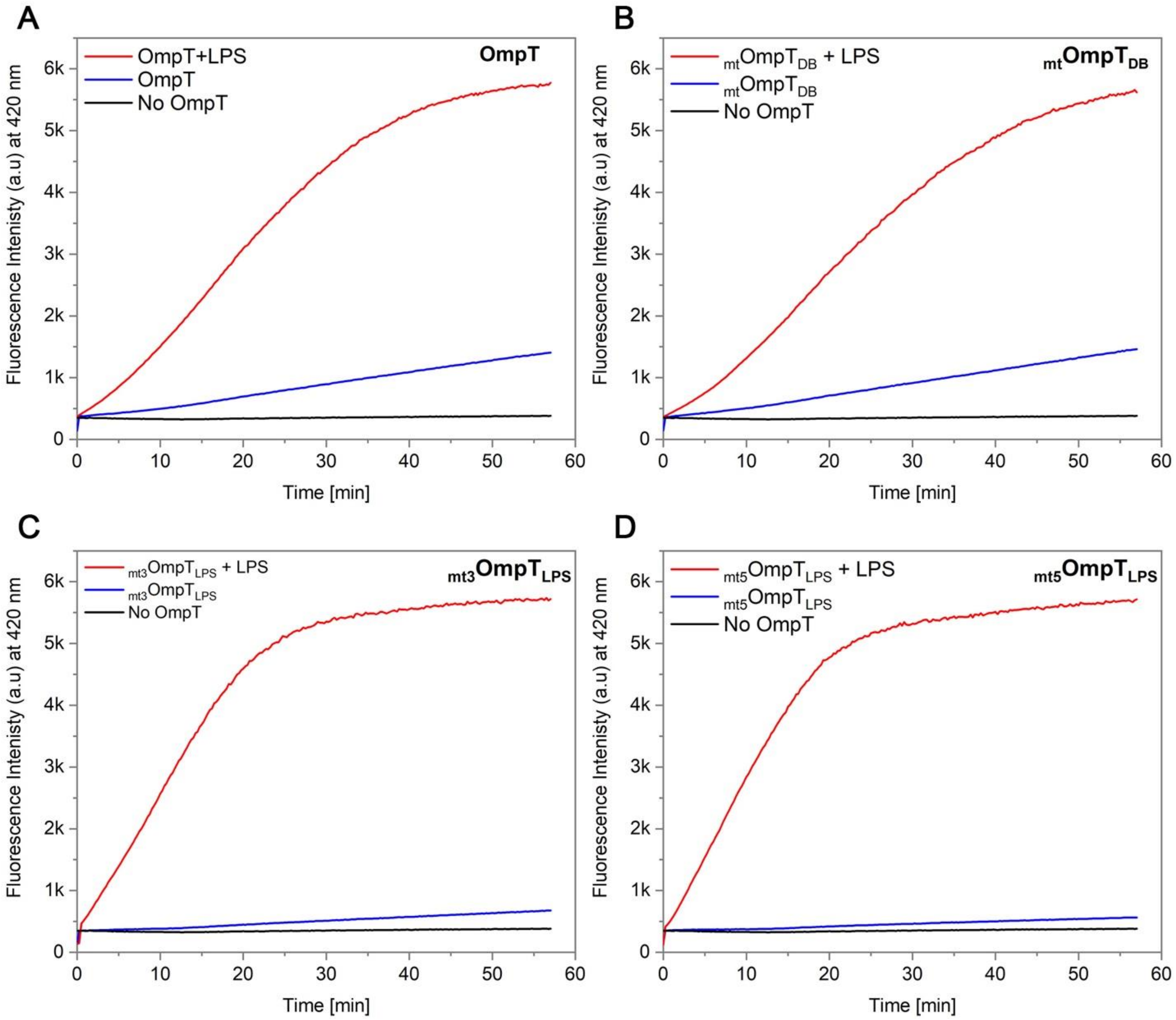

Figure S4. OmpT protease activity in the presence and absence of externally added LPS. FRET based assay was used to measure protease activity of (A) wild type OmpT, (B) $_{mt}$OmpT$_{DB}$, (C) $_{mt3}$OmpT$_{LPS}$, (D) $_{mt5}$OmpT$_{LPS}$ in the presence (red) and absence (blue) of LPS. Black lines indicate fluorescence intensity of control samples containing FRET peptide with LPS, but no OmpT added.

References

1. Rath, A.; Glibowicka, M.; Nadeau, V.G.; Chen, G.; Deber, C.M. Detergent binding explains anomalous SDS-PAGE migration of membrane proteins. *Proc. Natl. Acad. Sci. U. S. A.* **2009**, *106*, 1760–1765.
2. Ohnishi, S.; Kameyama, K.; Takagi, T. Characterization of a heat modifiable protein, Escherichia coli outer membrane protein OmpA in binary surfactant system of sodium dodecyl sulfate and octylglucoside. *Biochim. Biophys. Acta - Biomembr.* **1998**, *1375*, 101–109.
3. Dornmair, K.; Kiefer, H.; Jähnig, F. Refolding of an integral membrane protein; OmpA of Escherichia coli. *J. Biol. Chem.* **1990**, *265*, 18907–18911.
4. GARTEN, W.; HINDENNACH, I.; HENNING, U. The Major Proteins of the Escherichia coli Outer Cell Envelope Membrane: Characterization of Proteins II* and III, Comparison of All Proteins. *Eur. J. Biochem.* **1975**, *59*, 215–221.
5. Kramer, R.A.; Zandwijken, D.; Egmond, M.R.; Dekker, N. In vitro folding, purification and characterization of

Escherichia coli outer membrane protease OmpT. *Eur. J. Biochem.* **2000**, *267*, 885–893.
6. Visudtiphole, V.; Thomas, M.B.; Chalton, D.A.; Lakey, J.H. Refolding of Escherichia coli outer membrane protein F in detergent creates LPS-free trimers and asymmetric dimers. *Biochem. J.* **2005**, *392*, 375–81.
7. Hong, H.; Tamm, L.K. Elastic coupling of integral membrane protein stability to lipid bilayer forces. *Proc. Natl. Acad. Sci.* **2004**, *101*, 4065–4070.
8. Wessel, D.; Flügge, U.I. A method for the quantitative recovery of protein in dilute solution in the presence of detergents and lipids. *Anal. Biochem.* **1984**, *138*, 141–143.
